# Real-Time Rainfall Estimation Using Microwave Links: A Case Study in East China during the Plum Rain Season in 2020

**DOI:** 10.3390/s21030858

**Published:** 2021-01-28

**Authors:** Kun Song, Xichuan Liu, Taichang Gao

**Affiliations:** College of Meteorology and Oceanography, National University of Defense Technology, Changsha 410073, China; songkun_0521@foxmail.com (K.S.); 2009gaotc@gmail.com (T.G.)

**Keywords:** rain sensor, microwave link, rainfall estimation

## Abstract

Accurate and real-time rainfall estimation is a pressing need for forecasting the flood disaster and reducing the loss. In this study, we exploit the potential of estimating the rainfall by microwave links in East China. Eight microwave links at 15 GHz and 23 GHz, operated by China Mobile, are used for estimating the rain rate in real-time in Jiangyin, China from June to July 2020. First, we analyze the correlation between the rain-induced attenuation of microwave links and the rain rate measured by rain gauges. The correlation coefficient values are higher than 0.77 with the highest one over 0.9, showing a strong positive correlation. The real-time results indicate that microwave links estimate the rainfall with a higher temporal resolution than the rain gauges. Meanwhile, the rain rate that was estimated by microwave links also correlates well with the actual rain rate, and most of the values of the mean absolute error are less than 1.50 mm/h. Besides, the total rainfall’s relative deviation values are less than 5% with the smallest one reaching 1%. The quantitative results also indicate that microwave links could lead to better forecasting of water levels and, hence, better warnings for flood disasters.

## 1. Introduction

Accurate and real-time rainfall estimation is essential for forecasting flood disaster and reducing the loss of life and property. At present, the rain gauge (RG) is considered to be an accurate ground-based estimation. However, it does not provide rain data with high spatial resolution [1,2]. The weather radar can make up for RG’s shortcoming, but ground clutter often affects it, which leads to less accurate ground-level measurements [3]. Therefore, it is necessary to develop an accurate, real-time, and ground-based approach to be a complement to the RG and the weather radar.

Rainfall estimation using the widespread commercial microwave links (CML) has become a promising approach in recent years, because CML’s carrier signal over 10 GHz is attenuated by the rainfall. The rain-induced attenuation can be recorded as the reference signal level (RSL) with the rainfall information [4,5]. Therefore, rainy/non-rainy periods can be classified by the attenuation threshold [6], Markov switching model [7], and extension of the Multifamily Likelihood Ratio Test [8]. Additionally, 2 GHz-CML also can classify rainy and non-rainy with the combination of statistical parameters of the attenuation [9]. The classification accuracy of the studies above is higher than 80%, indicating a satisfactory performance. Further, rainfall estimation by CML and uncertainty due to spatial rainfall variability has been studied [10]. In 2010, the near-surface rain rate in the alpine region of Southern Germany has been estimated by CMLs, and the result positively correlates with the rainfall being estimated by radars [11]. A rainfall monitoring in Burkina Faso, in Sahelian West Africa was conducted in 2012 with the correlation of the result reaching 0.8 [12]. Besides, rainfall maps have been reconstructed in Israel by the network of CMLs since 2005 [13,14]. Similarly, regional rainfall in the Netherlands has been estimated in real-time by over 2400 CMLs since 2011 [15]. Sufficient conditions for reconstructing rainfall maps have been given by characterizing the statistics of the measurements [16]. Rainfall estimation by CML has two significant advantages: high spatial resolution due to widespread CMLs and low cost due to no extra instruments [17,18].

In this study, we estimate the rainfall by CMLs during the plum rain season in 2020 in Jiangyin, China. The average total rainfall of the period in South China is 436.3 mm, which is the second-highest rainfall for the same time since 1961 [19]. This study is the first CML-based rainfall estimation in China. When compared with previous studies, we focus more on the urban street’s rain rate, which is a smaller scale rainfall estimation. Thus, we use eight CMLs (less than 1 km) to estimate rainfall. The rain-induced attenuation is extracted from RSL. Subsequently, we analyze the correlation between the rain-induced attenuation and rain rate estimated by RG. Besides, we compare the rainfall estimation by different methods and then discuss the influence of the frequency and the length of CML. The advantages and limitations of this approach are also pointed in this study.

## 2. Method for Estimating the Path-Averaged Rain Rate by CML

The signal of CML is attenuated by raindrops in its transmission path. An empirical power–law equation between specific attenuation due to rain and the rain rate can be written, as follows [20,21]:

(1) $$k=a\cdot R^{b}$$

where *k* (dB/km) is specific attenuation due to rainfall, *R* (mm/h) is an equivalent path-averaged rain rate of CML transmission path (because rainfall is not uniform across the path in reality), and *a* and *b* are the coefficients. *R* can be retrieved by the following equation on condition that *k* is known [21]:

(2) $$R={\left(\frac{k}{a}\right)}^{\frac{1}{b}}$$

In this study, the values of *a* and *b* are referred to Reference [21]. The total attenuation of CML is given by Equation (3) [8]:

(3) $$A_{t}\left(t\right)=A_{p}\left(t\right)+A_{R}\left(t\right)+A_{w}\left(t\right)+A_{a}\left(t\right)+e$$

where *A_t_*(*t*) (dB) is the total attenuation of CML at time *t*; *A_p_*(*t*) (dB) is the free path loss of the attenuation at time *t*; *A_R_*(*t*) (dB) is the rain-induced attenuation of CML at time *t*; *A_w_*(*t*) (dB) is the water vapor that is induced attenuation at time *t*; *A_a_*(*t*) (dB) is the attenuation by the air (oxygen mainly) at time *t*; and, *e* (dB) is the measurement noise of CML. For simplification, Equation (3) is rewritten, as follows:

(4) $$A_{t}\left(t\right)=A_{R}\left(t\right)+baseline$$

where the baseline represents the attenuation due to other than rain at time *t*. Therefore, it is necessary to extract the baseline from *A_t_*(*t*). In general, the baseline is considered to be either piecewise constant [5] or slowly changing with time [22]. However, in reality, the baseline will be continuously updated with the minimum attenuation being measured in the past few minutes. [23]. We set Δ*T* to represent the past few minutes. Thus, the baseline is determined by Equation (5) in this study:

(5) $$baseline=\min\left\{A_{t}\left(t_{1}\right),A_{t}\left(t_{2}\right),\cdots ,A_{t}\left(t_{N}\right)\right\}$$

where *t*_1_, *t*_2_, …, *t_N_* belongs to Δ*T*. We set Δ*T* = 15 min in this study. Subsequently, we can extract *A_R_*(*t*) from *A_t_*(*t*) by Equation (4). Additionally, the relationship between *A_R_*(*t*) (dB) and *k* (dB/km) is as follows:

(6) $$A_{R}\left(t\right)=k\cdot l$$

where *l* (km) is the length of CML.

Finally, input *A_R_*(*t*) into Equation (2), and the path-averaged rain rate can be calculated, as follows:

(7) $$R={\left(\frac{A_{R}\left(t\right)}{a\cdot l}\right)}^{\frac{1}{b}}$$

## 3. Data

**Figure 1:**
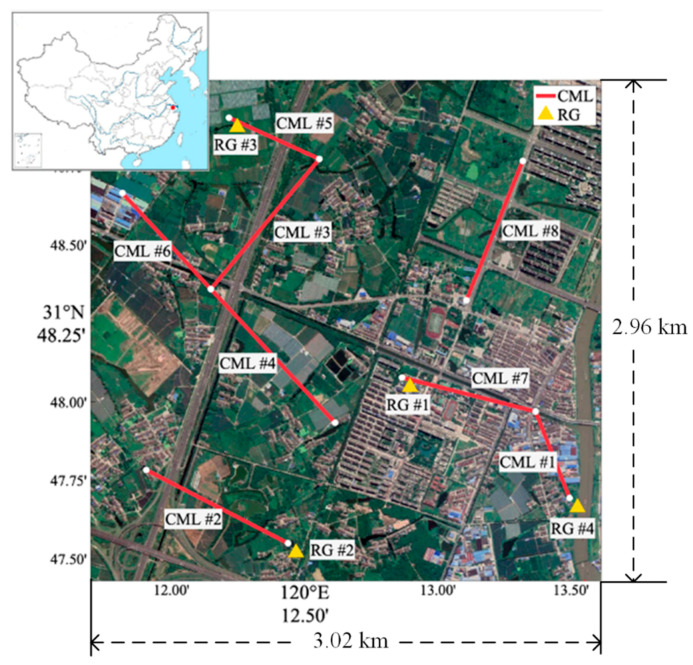
Locations of commercial microwave links (CMLs) and rain gauges (RGs) in the experimental area. The red point of the upper left subfigure shows the location of Jiangyin in China.

In this study, eight horizontal polarization CMLs at 15 GHz and 23 GHz are used to estimate the rain rate in Jiangyin, China (from 10 June to 7 July 2020). They were installed on the base towers over 30 m (operated by China Mobile) and provide RSL with a resolution of 0.1 dB and a sample time of one minute. Table 1 shows the frequency, length, and power–law coefficients. Figure 1 shows the locations of eight CMLs and four tipping-bucket RGs (0.5-mm resolution and five-minute sample time). RGs are installed near CMLs to verify the accuracy of rainfall estimation.

**Figure 2:**
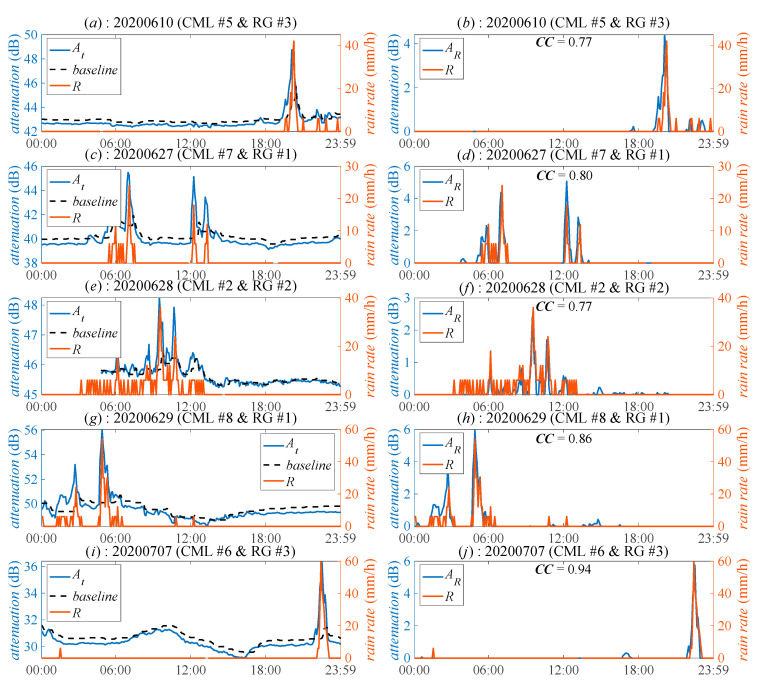
Time series of the total attenuation (*A_t_*), the baseline, the rain-induced attenuation (*A_R_*), and the rain rate (*R*) in different days by different CMLs. (**a**,**c**,**e**,**g**,**i**) show the comparison among *A_t_*, *baseline*, and *R*; (**b**,**d**,**f**,**h**,**j**) show the comparison between *A_R_*, and *R*.

Ten days RSLs of CMLs are used in this study. The total rainfall, as measured by different RGs, ranges from 217.5 mm to 237.5 mm with the highest daily rainfall over 40 mm. Figure 2a,c,e show the comparison among *A_t_*, *baseline* of CML, and *R* of RG; Figure 2b,d,f show the comparison between *A_R_* and *R*. It shows that *A_t_* and *A_R_* both positively correlate with *R*. The correlation coefficients (*CC*) between *A_R_* and *R* are higher than 0.7 with the highest one over 0.9, indicating that CMLs have the potential of estimating the rainfall.

## 4. Results

We calculate the mean absolute error (*MAE*), absolute deviation (*AD*), and relative deviation (*RD*) to quantify the results. Equations are shown, as follows:

(8) $$MAE=\frac{1}{n}\sum_{i=1}^{n}\left|R_{M,i}-R_{R,i}\right|$$

(9) $$AD=Rainfall_{M}-Rainfall_{R}$$

(10) $$RD=\frac{Rainfall_{M}-Rainfall_{R}}{Rainfall_{R}}\cdot 100\%$$

where *R_M,i_* and *R_R,i_* represent the rain rate by CML and by RG at time index *i*, respectively; and, *Rainfall_M_* and *Rainfall_R_* represent the total rainfall by CML and RG, respectively.

**Figure 3:**
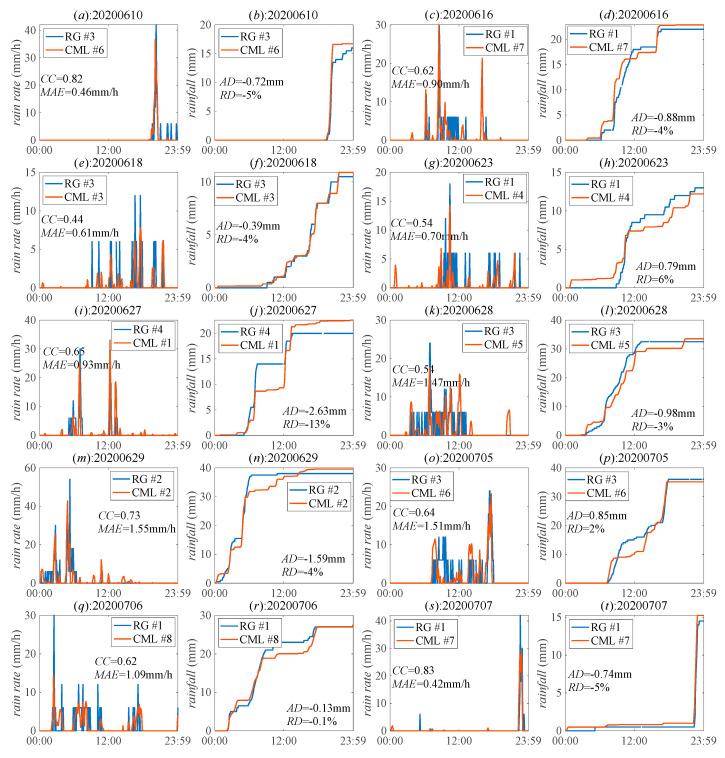
Result of the rain rate and rainfall by CMLs and RGs on different days. Subfigures in the first and the third column show the results of the rain rate, and subfigures in other columns show the result of the rainfall.

Figure 3 shows the results of the rain rate and daily rainfall. The *MAE* values are less than 1.6 mm/h, and most of the *CC* values are higher than 0.6 with the highest value reaching to 0.83. It indicates that CML accurately estimates the rain rate. Besides, most of the *RD* values for daily rainfall are less than 10%, which means that CML can also estimate the daily rainfall.

**Figure 4:**
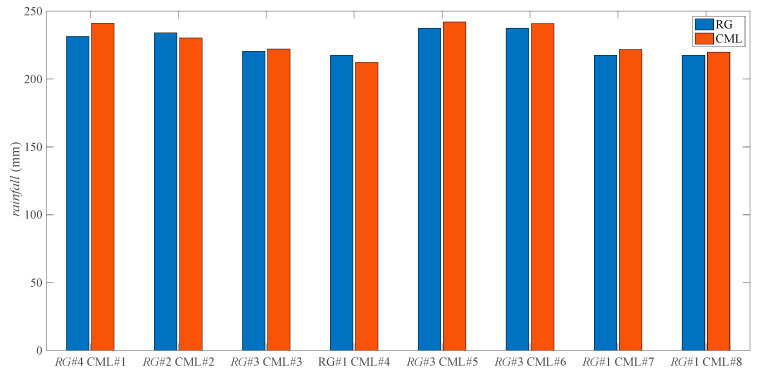
Comparison between the total rainfall of ten days estimated by eight CMLs and that by RGs near the CMLs.

Figure 4 shows the comparison of the total rainfall between CML and RG, with Table 2 providing the quantitative results. It shows that the *MAE* values are less than 1.2 mm/h, and most of the *RD* values are less than 5% with the smallest one reaching 1%, indicating an accurate estimation by CML. Note that CML estimation is the path-averaged measurement, while RG estimation is the point-measurement, which suggests that the results by the two estimations are not entirely consistent with each other.

## 5. Discussions

### 5.1. Influence of the Frequency and the Length of CML

CML at a higher frequency has a larger attenuation signal under the same weather condition. A different strength of attenuation signals can influence the accuracy of rainfall estimation [10,24]. Estimations of the rain rate by CML #5–8 (at 23 GHz) are more accurate than CML #1–4 (at 15 GHz) in Table 2, which implies that rainfall estimation by CML at a higher frequency is more suitable for estimating the rainfall. It is because the extraction of the rain-induced attenuation from a higher attenuation is more accurate [10].

A study has investigated the impact on RSL of short CMLs caused by the near-field effect, showing that the links shorter than 1.3 km have poorer performance by the power–law equation [25]. A recurrent neural network approach was used to overcome the effect. In our study, CML #1 and #5 (shorter than 0.6 km) also performed in a similar manner as in Table 2, indicating that the near-field effect influences the rainfall estimation. Consequently, we will adopt the deep-learning method to solve the problem according to Reference [25] in the future.

### 5.2. Advantages and Limitations of the Rainfall Estimation by CMLs

**Figure 5:**
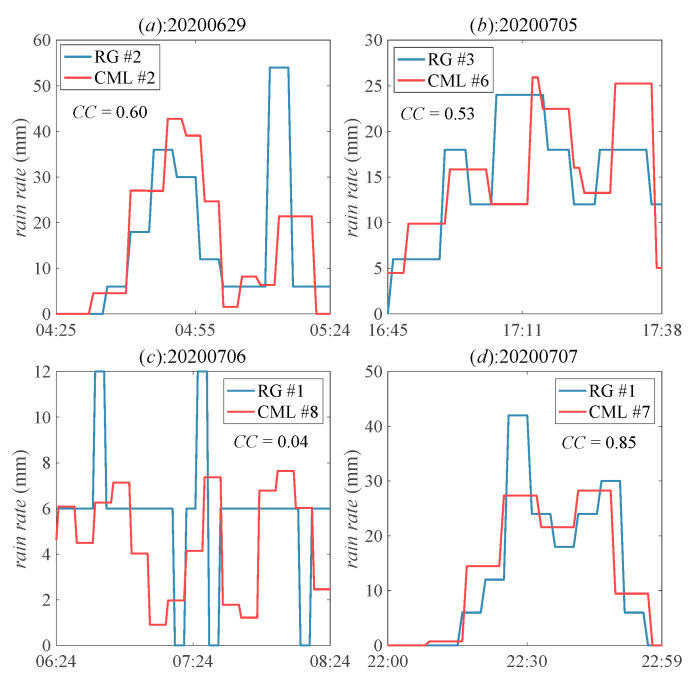
Comparison of the rain rate estimation with the one-minute sample time by the CMLs and that by RGs with the five-minute sample time on different days. The *CC* values in (**a**,**b**,**d**) are better than the value in (**c**).

The sample time of CML and RG are one-minute and five-minute, respectively in our study. Figure 5 gives the comparison of the rain rate estimation between CML and RG. It shows that CML can sense the rain rate more constantly than RG, which is necessary in ensuring the life safety during the rainstorm. We assume that the rain rate by RG during the five-minute interval is constant in order to analyze the correlation of one-minute data. The *CC* values in Figure 5a,b,d are acceptable, but the value in Figure 5c is not good, because the rainfall estimation by CML is the path-averaged, while the estimation by RG is the point measurement. Besides, the rain rate is time-varied in reality; however, we assume the estimation by RG as a constant during the sample interval, which increases the correlation error. The resolutions of CMLs at 15 GHz and 23 GHz are 1.2 mm/h and 0.4 mm/h, respectively, with the quantization level of 0.1 dB, which are higher than that of RG (6 mm/h), according to Equation (4). This shows that CMLs can more accurately estimate the rainfall rate.

However, when compared with the dedicated instrument for estimating the rainfall, CML is uncertain, because their geometry and frequencies are optimized in order to improve communication performance rather than estimate the rainfall [26,27,28]. Many error sources, such as the uncertainty of the rain-induced attenuation model, the baseline determination, and the antenna wetting, influence the accuracy of the rainfall estimation [10]. Many publications studied the error sources to improve the accuracy [29,30,31].

### 5.3. Related Work

The rain rate of one-minute resolution has been estimated by using a German-wide CMLs with the average bias of the rainfall estimation reaching to 19% (less than 2% in our study) [32]. Most CMLs, which range from 10 to 40 GHz, have a length from 5 to 10 km. When compared with Reference [32], the lengths of CMLs in our study are less than 1 km, because we focus more on the rain rate of the street scale, and the rain rate by short CML is more representative for the smaller scale. In 2017, an experiment of rainfall estimation with 10 s sample time has been conducted using a 700 m microwave link in China [33]. The results showed that correlations of rainfall estimation varied from 0.6 to 0.9, which is similar to our study. Besides, only 2.5% of RSL during the non-rainy time was counted as rainy time. Unlike Reference [33], eight microwave links in our study are operated by China Mobile for commercial communication with less estimation accuracy. It is the first CML-based rainfall estimation in China.

## 6. Conclusions

In this study, eight CMLs at 15 GHz and 23 GHz are used to estimate the rainfall, in Jiangyin, China, during the plum rain season in 2020. We adopt a dynamic determination of the baseline in order to continuously calculate the baseline for reducing the uncertainties and obtaining the accurate rain-induced attenuation. Subsequently, we analyze the correlation between *A_R_* and *R*. The *CC* values are higher than 0.77, with the highest one reaching to 0.94, indicating a strongly positive correlation, as shown in Figure 3.

Besides, the quantitative results of the rainfall show that most of the *MAE* values are less than 1.50 mm/h. The *RD* values are less than 5%, with the smallest one reaching 1%, which indicated that CML can accurately estimate the rainfall. Additionally, we discuss the influence of the frequency and the advantages of CML. This study is also compared with the previous works.

This study verifies that CML can be a complement to the weather radar and the rain gauge. Further, the CML system could provide much more refined rain data for meteorology, hydrology, and many other fields if cooperating with the mobile network operators. Of course, further works need to be done due to the limitations of the CML.

## Figures and Tables

**Table 1:** The frequencies and lengths of eight CMLs. *f* and *l* represent the frequency and the length.

CML	#1	#2	#3	#4	#5	#6	#7	#8
*f* (GHz)	15	15	15	15	23	23	23	23
*l* (km)	0.55	0.95	1.00	1.08	0.59	0.77	0.82	0.89
*a*	0.04	0.04	0.04	0.04	0.13	0.13	0.13	0.13
*b*	1.12	1.12	1.12	1.12	1.02	1.02	1.02	1.02

**Table 2:** The overall results of rainfall estimation by the CMLs.

CML	#1	#2	#3	#4	#5	#6	#7	#8
*MAE* (mm/h)	1.12	1.14	1.12	1.15	1.01	1.00	0.91	0.92
*AD* (mm)	9.5	−3.7	1.7	−5.2	4.6	3.4	4.3	2.4
*RD* (%)	4	−2	1	−2	2	1	2	1

## Data Availability

The data presented in this study are available on request from the corresponding author.
